# Breast cancer screening among Medicare Advantage enrollees with dementia

**DOI:** 10.1186/s12913-024-10740-7

**Published:** 2024-03-05

**Authors:** Eli Raver, Wendy Y. Xu, Jeah Jung, Sunmin Lee

**Affiliations:** 1 Division of Health Services Management and Policy, College of Public Health, The Ohio State University, Columbus, OH USA; 2 Department of Health Administration and Policy, College of Public Health, George Mason University, Fairfax, VA USA; 3 Department of Medicine, School of Medicine & Chao Family Comprehensive Cancer Center, University of California, Irvine, Irvine, CA USA

**Keywords:** Medicare Advantage, Breast cancer screening, Alzheimer’s disease and related dementias

## Abstract

**Background:**

The decision to screen for breast cancer among older adults with dementia is complex and must often be individualized, as these individuals have an elevated risk of harm from over-screening. Medicare beneficiaries with dementia are increasingly enrolling in Medicare Advantage plans, which typically promote receipt of preventive cancer screening among their enrollees. This study examined the utilization of breast cancer screening among Medicare enrollees with dementia, in Medicare Advantage and in fee-for-service Medicare.

**Methods:**

We conducted a pooled cross-sectional study of women with Alzheimer’s disease and related dementias or cognitive impairment who were eligible for mammogram screening. We used Medicare Current Beneficiary Survey data to identify utilization of biennial mammogram screening between 2012 and 2019. Poisson regression models were used to estimate prevalence ratios of mammogram utilization and to calculate adjusted mammogram rates for Medicare Advantage and fee-for-service Medicare enrollees with dementia, and further stratified by rurality and by dual eligibility for Medicare and Medicaid.

**Results:**

Mammogram utilization was 16% higher (Prevalence Ratio [PR] 1.16; 95% CI: 1.05, 1.29) among Medicare Advantage enrollees with dementia, compared to their counterparts in fee-for-service Medicare. Rural enrollees experienced no significant difference (PR 0.99; 95% CI: 0.72, 1.37) in mammogram use between Medicare Advantage and fee-for-service Medicare enrollees. Among urban enrollees, Medicare Advantage enrollment was associated with a 21% higher mammogram rate (PR 1.21; 95% CI: 1.09, 1.35). Dual-eligible Medicare Advantage enrollees had a 34% higher mammogram rate (PR 1.34; 95% CI: 1.10, 1.63) than dual-eligible fee-for-service Medicare enrollees. Among non-dual-eligible enrollees, adjusted mammogram rates were not significantly different (PR 1.11; 95% CI: 0.99, 1.24) between Medicare Advantage and fee-for-service Medicare enrollees.

**Conclusions:**

Medicare beneficiaries age 65–74 with Alzheimer’s disease and related dementias or cognitive impairment had a higher mammogram use rate when they were enrolled in Medicare Advantage plans compared to fee-for-service Medicare, especially when they were dual-eligible or lived in urban areas. However, some Medicare Advantage enrollees with Alzheimer’s disease and related dementias or cognitive impairment may have experienced over-screening for breast cancer.

**Supplementary Information:**

The online version contains supplementary material available at 10.1186/s12913-024-10740-7.

## Background

Alzheimer’s disease and related dementias (ADRD) are degenerative neurological conditions that result in memory loss, impaired cognition, and decision-making [1]. Today, 6.5 million Americans age 65 and older are living with ADRD, and this number is projected to grow to 14 million by 2060 [2]. Women are nearly twice as likely as men to be affected by ADRD [1]. The presence of ADRD poses challenges in managing health and activities of daily living for older adults.

Medicare is the primary payer for individuals with ADRD, which usually develops with age. In recent years, Medicare Advantage (MA), a private managed care alternative to traditional fee-for-service Medicare (FFS), experienced rapid enrollment growth. About 50% of Medicare beneficiaries are covered by MA plans in 2023 [3]. A growing number of Medicare beneficiaries with chronic conditions choose to enroll in MA plans [4]. In 2016, 6.5% of MA enrollees were diagnosed with ADRD [5]. The number of individuals with ADRD enrolling in MA will likely rise, as Medicare promotes flexible MA plan designs to serve beneficiaries with complex care needs [6].

MA plans are paid by capitation and are incentivized to enhance preventive services, such as screenings. MA plans are also required by the Centers for Medicare & Medicaid Services (CMS) to publicly report their enrollees’ receipt of preventive services, such as breast cancer screening recommended by the United States Preventive Services Task Force (USPSTF). While CMS recently revised screening quality metrics, starting with the 2020 Star Ratings, to exclude elderly enrollees with both frailty and advanced illness, including dementia [7], the mammography screening measure has been a major quality indicator of MA plans for many years [8]. Medicare offers bonus payments to MA plans for higher quality rating, which creates further financial incentives to increase breast cancer screening rates. As a result, MA plan enrollees generally have higher mammogram rates than FFS enrollees [8].

Older Medicare beneficiaries are increasingly enrolling in MA, and the risks for breast cancer and ADRD both increase with age [1, 9]. The USPSTF recommends biennial screening mammography for women aged 50 to 74 years without considering individuals with ADRD [10]. Based on this guidance, MA plans may similarly promote use of preventive mammograms for all of their eligible enrollees, regardless of ADRD status. However, providers that contract with managed care plans must balance the benefits and risks of ordering mammograms for older women with dementia, who are at an increased risk of the harms of over-screening, including over-diagnosis and over-treatment of breast cancer. This confluence of factors will make it increasingly important to understand mammogram utilization patterns among MA and FFS enrollees with ADRD. However, mammogram use among MA and FFS enrollees with ADRD is not known; filling this knowledge gap may help inform clinical guidelines and Medicare policies for MA plans.

Individuals with ADRD and comorbid breast cancer can experience poor cancer-related outcomes, including late-stage diagnosis, limited treatment options, increased mortality, and high costs [11, 12]. Early detection of breast cancer through preventive screening provides an opportunity for patients and caregivers to plan for a preferred course of therapy at an earlier disease stage, avoiding invasive treatments [13]. However, it may be challenging to perform screening tests and further diagnostic workup for elderly patients with cognitive issues, as these procedures can cause discomfort, confusion, and even fear. Routine mammograms may also result in false positives and additional expensive procedures [10, 13–15].

This discussion suggests that cancer screening decisions can be complex for patients with ADRD or any cognitive issues. However, little is known about the use of cancer screenings by patients with ADRD or cognitive impairment in MA plans that promote preventive screening use. Our study fills this gap and examines utilization of breast cancer screening among patients with ADRD or cognitive impairment in MA relative to FFS. It thus helps us understand how MA plans manage utilization of cancer screening tests in situations where the decision of whether to receive those tests is complex.

## Methods

### Data

We conducted a retrospective cross-sectional study using 2012–2019 Medicare Current Beneficiary Survey (MCBS) data [16]. This nationally representative survey collects information about Medicare beneficiaries such as their health conditions, health care utilization and spending, and beneficiary and household socio-demographic characteristics. The MCBS uses a short, rotating panel design where participants may be interviewed multiple times over a four-year period. MCBS data for 2014 are not available due to a redesign of the survey during that year. MA versus FFS enrollment information from Medicare administrative records is added to MCBS. The Ohio State University Institutional Review Board approved this study.

### Population studied

Our study sample included women aged 65–74 years who were recommended to receive biennial mammogram screening according to the USPSTF. We focused on community-dwelling individuals who had ADRD or cognitive impairment, which often precedes a formal diagnosis of ADRD [17]. Cognitive impairment includes difficulty in concentrating, remembering, or making decisions that interferes with daily activities [18]. The MCBS survey asks if beneficiaries had a history of Alzheimer’s disease, other dementia, or any component of cognitive impairment. Individuals with a history of breast cancer were excluded. Survey participants may designate a proxy respondent to answer survey questions on their behalf, if they are unable to complete the survey or have difficulty answering specific questions, as in the case of severe memory loss. Proxies are individuals who are familiar with the survey participant’s health status and health care utilization, and they are typically a caregiver, spouse, or family member [19].

The study sample was further limited to beneficiaries who enrolled in either MA or FFS for a full calendar year. We excluded enrollees who switched between FFS and MA mid-year (2.2% of the sample).

### Measures

The study outcome is the receipt of breast cancer screening. We constructed this measure based on the USPSTF recommendations that were in effect during the study period. From 2012 to 2019, the USPSTF recommended that women aged 50–74 years receive a biennial mammogram screening [10, 20]. The MCBS asks women whether they received a mammogram within the last year. We considered beneficiaries as following the recommendation if they reported receiving a mammogram in either of two consecutive survey years.

### Statistical analysis

We calculated descriptive statistics of the sample characteristics and unadjusted mammogram rates, using MCBS analytic weights. We then performed Poisson regression to compare breast cancer screening utilization between MA and FFS, controlling for individual characteristics. Cross-sectional analysis using Poisson regression estimates prevalence ratios of the outcome. When the outcome (i.e., mammogram screening) is prevalent (> 10%), directly estimating prevalence ratios is preferable to odds ratios, which overestimate the strength of association between the predictor and outcome [21, 22]. We further calculated the regression-adjusted mean mammogram utilization rates in each MA and FFS group. For the Poisson regression analyses and regression-adjusted means, we used inverse probability of treatment weighting to balance observed characteristics between MA and FFS enrollees, and assessed the covariate balance after weighting via absolute standardized mean differences between enrollment groups [23, 24]. Following MCBS recommendations, we used the survey analytic weights and Fay’s method of balanced repeated replications for variance estimation to account for the complex survey design of MCBS, including stratified and cluster sampling, and the panel design where individuals are interviewed more than once [19].

The control variables included age, self-reported race/ethnicity, comorbid conditions (excluding ADRD or cognitive impairment), education, income, rurality, and dual-eligibility. Rurality was defined as residence in a non-metropolitan area, following the Rural Urban Commuting Area classification codes [25]. Dual eligibility was defined as any eligibility for both Medicare and Medicaid for any part of the year.

We also examined how the association between MA enrollment and mammogram utilization differs across patient subgroups for whom access challenges have been underscored in the literature about MA [26, 27]. Mammogram utilization may differ by the rurality of patient residence, as rural and urban areas have differing levels of health care resources and accessibility of primary care and imaging services [28]. We thus performed stratified analyses to examine differences in mammogram use between MA and FFS by rurality of patient residence. Similarly, Medicare beneficiaries who are dually eligible for Medicaid typically have lower income and greater health care needs than non-dual-eligible Medicare beneficiaries [29]. We estimated separate Poisson regressions for dual-eligible and non-dual-eligible enrollees. For each subgroup analysis, we calculated prevalence ratios of MA versus FFS mammogram use, as well as the regression-adjusted mammogram utilization rates.

We performed two sensitivity analyses. First, we repeated the Poisson regression model to compare breast cancer screening rates among MA and FFS enrollees age 75 years and older. For this age group, during the study period, the USPSTF concluded that there was insufficient evidence to assess the benefits and harms of screening mammography [10, 20]. Therefore, breast cancer screenings should not be promoted among MA enrollees age 75 and older. If MA would present higher rates of mammogram utilization than FFS in this population, that would indicate over-screening in MA. Second, we estimated a regression model with an added indicator of using a proxy survey respondent. Dementia and cognitive impairment can affect self-reported screening use because of potentially impaired recall. To the extent that individuals with severe recall impairment use proxies for their interviews, including the indicator of proxy use in the model mitigates the impact of the recall issue on the results.

## Results

The study sample included 2,090 person-year observations. MA enrollees comprised 30.4% of the sample, the mean (SD) age was 69.4 (2.8) years, 23.6% of beneficiaries lived in rural areas, and 28.7% of beneficiaries were dually eligible for Medicare and Medicaid. Detailed sample characteristics are shown in Table 1.

**Table 1:** Sample characteristics and unadjusted mammogram rates^a^

	Sample Characteristics, % or mean (SD)		Unadjusted Mammogram Rate,^b^ %	
	Fee-for-Service Medicare	Medicare Advantage	Fee-for-Service Medicare	Medicare Advantage
Overall	--	--	51.3	61.5
Age in years, mean (SD)	69.3 (2.8)	69.6 (2.7)	--	--
Race / ethnicity	--	--	--	--
White (non-Hispanic), %	79.1	75.8	48.8	60.1
Black (non-Hispanic), %	11.5	14.8	60.6	67.2
Hispanic (any race), %	3.1	6.5	65.9	70.9
Asian, Native American, or other race (non-Hispanic), %	6.3	3.0	58.5	48.8
Urban residence, %	72.3	86.0	53.9	64.7
Rural residence, %	27.8	14.0	44.6	42.4
Dual-eligible, %	27.7	30.9	41.9	58.6
Non-dual-eligible, %	72.3	69.1	54.9	62.9
Number of body systems affected by comorbid conditions,^c^ mean (SD)	4.8 (1.7)	4.7 (1.6)	--	--
Cardiovascular disorder, %	81.9	84.5	50.1	62.0
Endocrine or metabolic disorder, %	84.6	85.2	52.0	62.8
Mental disorder, %	59.2	61.3	51.6	62.0
Musculoskeletal disorder, %	74.7	75.8	54.3	64.3
Neurological disorder, %	7.7	5.4	52.9	67.0
Respiratory disorder, %	32.7	32.0	48.0	59.2
Cancer, %	26.5	23.1	53.3	62.4
Vision impairment, %	56.2	54.9	50.5	60.3
Hearing impairment, %	54.6	52.6	52.7	61.4
Alzheimer’s disease and related dementias, %	11.3	12.6	47.4	58.9
Cognitive impairment, %	88.7	87.4	51.8	61.9
Income	--	--	--	--
Less than $15,000, %	31.7	33.6	41.5	60.6
$15,000 to $24,999, %	20.1	21.9	47.6	58.4
$25,000 to $49,999, %	25.7	27.1	57.8	59.1
$50,000 or more, %	22.5	17.3	60.9	71.2
Education	--	--	--	--
High school diploma or less, %	52.4	57.4	49.0	61.5
Vocational, technical, or business training, some college, or associate’s degree, %	30.6	27.6	49.5	56.4
Bachelor’s degree or higher, %	17.1	15.0	61.4	71.2
Survey respondents, n	1168	470	--	--
Observations (person-year), n	1476	614	--	--

Notes: ^a^Sample characteristics and unadjusted mammogram rates are weighted using Medicare Current Beneficiary Survey analytic weights

^b^The unadjusted mammogram rates are the proportion of observations in each enrollee characteristic group who reported receiving a biennial mammogram

^c^Comorbid conditions include self-reported cardiovascular disorders (arrhythmias, arteriosclerosis, coronary heart disease, heart failure, heart valve diseases, hypertension, or history of stroke), endocrine or metabolic disorders (diabetes, hyperlipidemia, or obesity), mental disorders (anxiety, depression, or other psychiatric disorder), musculoskeletal disorders (arthritis of any type, osteoporosis, or history of a broken hip), neurological disorders (Parkinson’s disease or paralysis) excluding Alzheimer’s disease and related dementias and cognitive impairment, respiratory disorders (asthma or chronic obstructive pulmonary disease), cancer (any type excluding breast cancer), vision impairment, and hearing impairment

The regression results (Table 2) showed that MA enrollment was significantly associated with higher mammogram utilization (Prevalence Ratio [PR] 1.16; 95% CI: 1.05, 1.29), compared to FFS enrollment. The adjusted rate of biennial mammogram screening was 60.0% (95% CI: 54.9, 65.0) among MA enrollees with ADRD or cognitive impairment, compared to 51.5% (95% CI: 48.5, 54.4) for their counterparts in FFS (results not shown).

Estimates of some covariates that captured demographic and socioeconomic characteristics are also worth mentioning. Compared to non-Hispanic White enrollees, both Black enrollees (PR 1.26; 95% CI: 1.13, 1.42) and Hispanic enrollees (PR 1.32; 95% CI: 1.10, 1.59) had higher mammogram utilization, although there was no significant difference for enrollees who were Asian, Native American, or another race (PR 1.13; 95% CI: 0.87, 1.46). Enrollees with lower income tended to have lower rates of mammogram screening. Enrollees with an annual income less than $15,000 had a mammogram utilization rate that was lower (PR 0.78; 95% CI: 0.67, 0.90) than those with an income of $50,000 or greater.

**Table 2:** Poisson regression model of mammogram utilization among Medicare enrollees age 65–74 with Alzheimer’s disease and related dementias or cognitive impairment

	Overall Sample (*n* = 2090)	
	Prevalence Ratio	95% CI
Medicare enrollment (ref: fee-for-service Medicare)		
Medicare Advantage	1.16	1.05, 1.29
Race/ethnicity (ref: White, non-Hispanic)		
Black	1.26	1.13, 1.42
Hispanic	1.32	1.10, 1.59
Asian, Native American, or other race	1.13	0.87, 1.46
Residence (ref: urban)		
Rural	0.78	0.70, 0.87
Age, years	1.00	0.98, 1.02
Number of body systems affected by comorbid conditions	1.03	1.00, 1.06
Dual eligibility (ref: no)		
Yes	0.91	0.79, 1.04
Income (ref: $50,000 or more)		
Less than $15,000	0.78	0.67, 0.90
$15,000 to $24,999	0.79	0.69, 0.91
$25,000 to $49,999	0.91	0.80, 1.02
Education (ref: Bachelor’s degree or higher)		
High school diploma or less	0.91	0.79, 1.03
Vocational, technical, or business training, some college, or associate’s degree	0.84	0.72, 0.99

Table 3 shows the stratified regression results for rural and urban enrollees. There was no significant difference in mammogram use between MA and FFS among rural enrollees (PR 0.99; 95% CI: 0.72, 1.37). Yet among urban enrollees, MA enrollment was associated with higher mammogram rates (PR 1.21; 95% CI: 1.09, 1.35).

**Table 3:** Poisson regression model of mammogram utilization among rural and urban Medicare enrollees with Alzheimer’s disease and related dementias or cognitive impairment

	Rural (*n* = 603)		Urban (*n* = 1487)	
	Prevalence Ratio	95% CI	Prevalence Ratio	95% CI
Medicare enrollment (ref: fee-for-service Medicare)				
Medicare Advantage	0.99	0.72, 1.37	1.21	1.09, 1.35
Race/ethnicity (ref: White, non-Hispanic)				
Black	1.32	0.98, 1.79	1.25	1.11, 1.42
Hispanic	1.41	0.59, 3.35	1.30	1.08, 1.57
Asian, Native American, or other race	1.07	0.25, 4.58	1.16	0.87, 1.55
Age, years	1.03	1.00, 1.07	1.00	0.98, 1.02
Number of body systems affected by comorbid conditions	1.04	0.97, 1.12	1.03	1.00, 1.06
Dual eligibility (ref: no)				
Yes	0.89	0.68, 1.16	0.92	0.78, 1.08
Income (ref: $50,000 or more)				
Less than $15,000	0.67	0.47, 1.02	0.80	0.68, 0.93
$15,000 to $24,999	0.57	0.42, 0.76	0.84	0.72, 0.98
$25,000 to $49,999	0.65	0.50, 0.83	0.95	0.83, 1.09
Education (ref: Bachelor’s degree or higher)				
High school diploma or less	0.83	0.59, 1.16	0.92	0.79, 1.06
Vocational, technical, or business training, some college, or associate’s degree	0.86	0.63, 1.18	0.82	0.68, 0.99

**Fig. 1:**
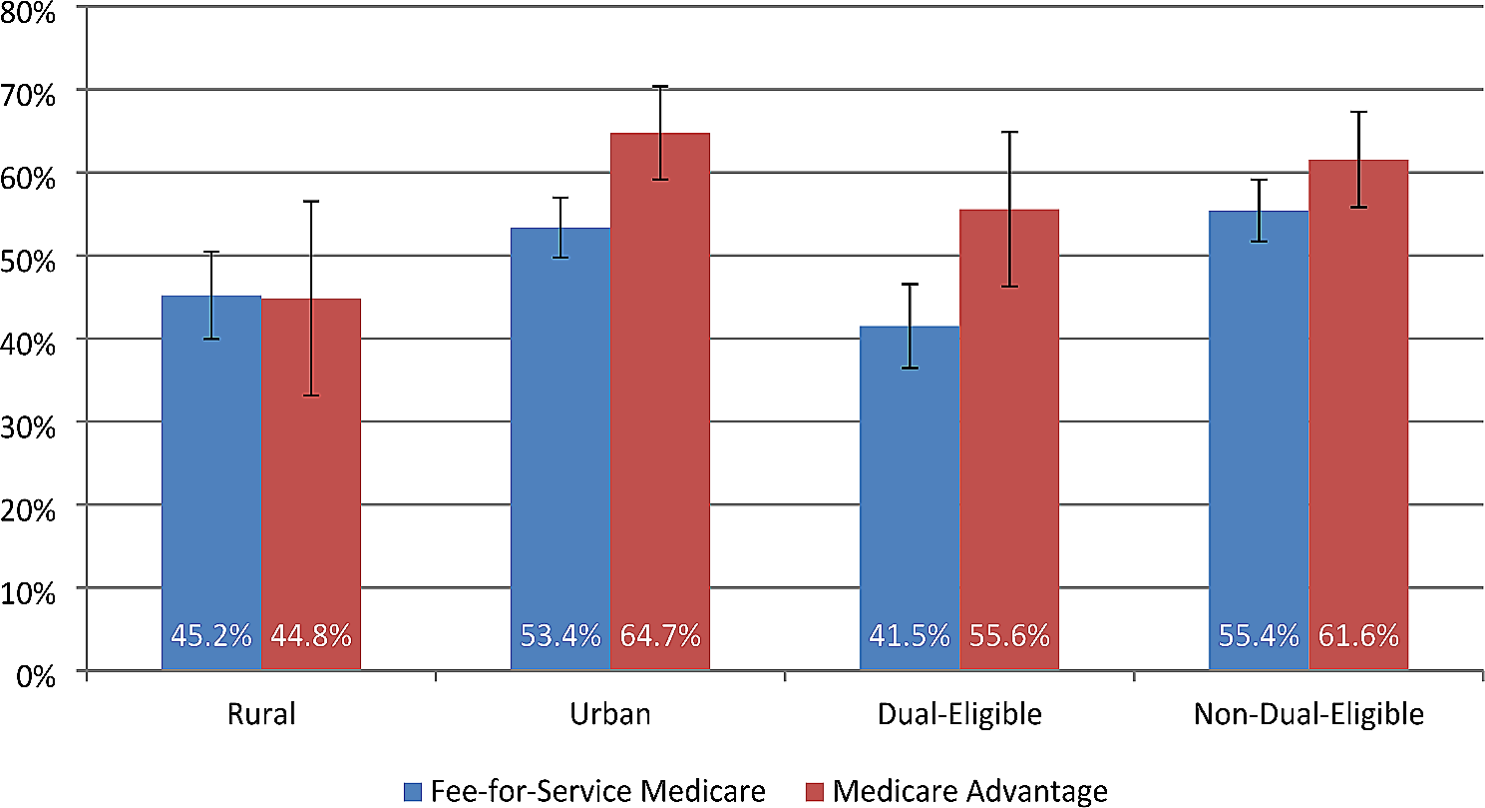
Adjusted biennial mammogram screening rates, stratified by rurality and by dual eligibility

Figure 1 displays adjusted average mammogram utilization rates for rural and urban enrollees by MA enrollment. The adjusted mammogram utilization rates were 44.8% (95% CI: 33.1, 56.5) for rural MA enrollees and 45.2% (95% CI: 40.0, 50.5) for rural FFS enrollees. The adjusted utilization rates were 64.7% (95% CI: 59.1, 70.4) among urban MA enrollees and 53.4% (95% CI: 49.8, 57.0) among their FFS counterparts.

Table 4 shows the stratified regression results for dual-eligible and non-dual-eligible enrollees, respectively. Among dual-eligible enrollees, those in MA had a higher mammogram screening rate than those in FFS (PR 1.34; 95% CI: 1.10, 1.63). Non-dual-eligible MA and FFS enrollees did not have significantly different mammogram utilization (PR 1.11; 95% CI: 0.99, 1.24).

**Table 4:** Poisson regression model of mammogram utilization among dual-eligible and non-dual-eligible Medicare enrollees with Alzheimer’s disease and related dementias or cognitive impairment

	Dual-eligible (*n* = 640)		Non-dual-eligible (*n* = 1450)	
	Prevalence Ratio	95% CI	Prevalence Ratio	95% CI
Medicare enrollment (ref: fee-for-service Medicare)				
Medicare Advantage	1.34	1.10, 1.63	1.11	0.99, 1.24
Race/ethnicity (ref: White, non-Hispanic)				
Black	1.26	1.01, 1.56	1.29	1.14, 1.46
Hispanic	1.21	0.88, 1.65	1.44	1.14, 1.82
Asian, Native American, or other race	1.11	0.74, 1.66	1.17	0.83, 1.66
Residence (ref: urban)				
Rural	0.70	0.56, 0.87	0.81	0.71, 0.92
Age, years	1.00	0.97, 1.04	1.00	0.98, 1.02
Number of body systems affected by comorbid conditions	1.06	1.00, 1.12	1.02	0.99, 1.06
Income (ref: $50,000 or more)				
Less than $15,000	0.53	0.41, 0.67	0.73	0.62, 0.88
$15,000 to $24,999	0.50	0.37, 0.67	0.80	0.69, 0.93
$25,000 to $49,999	0.40	0.23, 0.70	0.92	0.81, 1.04
Education (ref: Bachelor’s degree or higher)				
High school diploma or less	0.87	0.55, 1.37	0.90	0.78, 1.05
Vocational, technical, or business training, some college, or associate’s degree	0.70	0.41, 1.19	0.87	0.73, 1.03

As shown by Fig. 1, the adjusted mammogram rate among dual-eligible MA enrollees was 55.6% (95% CI: 46.3, 64.9), compared to 41.5% (95% CI: 36.5, 46.5) among dual-eligible FFS beneficiaries. Non-dual-eligible MA enrollees had an adjusted mammogram rate of 61.6% (95% CI: 55.8, 67.3), and the adjusted rate was 55.4% (95% CI: 51.7, 59.1) for non-dual-eligible FFS enrollees.

The full results of the sensitivity analysis among Medicare beneficiaries age 75 years and older (*n* = 4297) are displayed in the Supplemental File (Tables S1 and S2). There was no association between MA versus FFS enrollment and biennial mammogram screening among this group (PR 0.94, 95% CI: 0.84, 1.05). The adjusted mammogram rate was 33.7% (95% CI: 30.5, 37.0) among MA enrollees age 75 and older, and 35.8% (95% CI: 33.5, 38.2) among their FFS enrollee counterparts. Again, we found higher mammogram use rates among Black (PR 1.28, 95% CI: 1.10, 1.48) and Hispanic (PR 1.47, 95% CI: 1.12, 1.93) enrollees age 75 and older, compared to their non-Hispanic White counterparts.

Among the main study sample of Medicare beneficiaries age 65–74, 5.0% of MA enrollees and 1.8% of FFS enrollees used proxy survey respondents. The regression results from the analysis including an indicator for proxy respondent are presented in Supplemental Table S3. It produced similar results as the main analysis: MA enrollment was associated with higher use of mammogram, compared to FFS enrollment. Using a proxy respondent was not associated with reported mammogram use (PR 0.97; 95% CI: 0.77, 1.21).

## Discussion

ADRDs are predicted to become an increasingly large burden on the U.S. population and health care system [1]. We found that women with ADRD or cognitive impairment enrolled in MA plans were more likely to receive USPSTF-recommended preventive mammogram screening than their counterparts in FFS. This is consistent with the overall pattern of use of preventive services between MA and FFS [8, 30]. For MA enrollees age 65–74 with ADRD or cognitive impairment, the higher mammogram rates may partially represent an overuse of preventive breast cancer screening, among those whose health status does not warrant substantial benefits. Our analysis of beneficiaries age 75 and older, for whom breast cancer screening is not endorsed, showed no difference in mammogram rates between MA and FFS enrollees. This suggests that MA plans did not tend to promote preventive screening beyond broad guidelines more than TM.

The higher rates of mammogram use in MA than in FFS were observed among urban enrollees. However, there was no difference in mammogram use between MA and FFS among rural enrollees. In both MA and FFS, rural residents had much lower rates of mammogram use compared with urban residents. The differences in mammogram use between urban and rural areas appeared to be larger in MA than in FFS. MA enrollees in rural areas may encounter greater barriers to accessing preventive screenings, possibly due to limited provider networks formed by MA plans, compared with MA enrollees in urban areas. Alternatively, the chronic shortage of clinicians in rural areas may force providers to be more judicious with their time by attending to patients’ most pressing issues [31]. Therefore preventive screening for ADRD patients may become a lower priority than managing symptoms related to ADRD. Given the accelerated growth in MA enrollment in rural areas in recent years [32, 33], it will be important to continue monitoring patterns of preventive screenings among rural MA enrollees with cognitive challenges.

Dual-eligible MA enrollees had higher mammogram screening rates than dual-eligible FFS beneficiaries. Dual-eligible beneficiaries typically have more frequent encounters with the health care system, which may lead to more opportunities for providers to offer screening, especially with MA plans’ incentives [34]. Prior research found that dual-eligible enrollees in MA plans have greater access to primary care and higher preventive care utilization compared to their FFS counterparts [29]. Given the higher screening rates among dual-eligible beneficiaries with ADRD or cognitive impairment, our findings also suggest that further clinical tools are needed to help providers and patients with the discussion of potential harms of cancer screenings in the context of having ADRD or cognitive impairment, particularly considering patient quality of life and goals of care [14].

We also found that Black and Hispanic women with ADRD or cognitive impairment were more likely to receive mammograms than their White counterparts, even when mammograms are not recommended over age 75. This finding is consistent with recent literature that has shown similar differences and utilization patterns in the general population of women eligible for breast cancer screening [28]. Black and Hispanic women, along with their family and caregivers, may be more persistent in advocating for breast cancer screening, even in the face of advancing age and illness, particularly if they exhibit distrust of the health care system or clinical guidelines [35, 36]. It is also possible that Black and Hispanic women over-reported mammogram screenings at higher rates than White women, as found in self-reported surveys [37]. Regardless, Black and Hispanic women with breast cancer tend to be diagnosed at a later stage than White women, and Black women have higher mortality from breast cancer [38], indicating that the increased mammogram utilization does not necessarily translate to improved health outcomes for these patients. For Black and Hispanic individuals who also have ADRD, especially those over age 75, their increased breast cancer screening rates likely represent over-screening.

High breast cancer screening rates are generally considered an improvement in health care quality for most eligible individuals in the general population. However, the utility of cancer screening tests has been debated for individuals with ADRD. As the incidence of both ADRD and breast cancer increases with age, potential benefits and harms of preventive breast cancer screening can also increase with age [1, 10]. Mammogram screenings can help detect breast cancer at an early stage, but they could cause distress during the screening process to those with advancing age and declining cognitive functions [39]. Recent literature suggests that as individuals age, the mortality benefits of screening mammography become outweighed by harms, such as over-diagnosis [40, 41]. The addition of ADRD or cognitive impairment further complicates the decision to screen for breast cancer, and makes it even more likely for risks to outweigh benefits. This is especially concerning, as we observed more than a third of Medicare beneficiaries with ADRD or cognitive impairment in both MA and FFS continue to receive biennial mammograms after age 75.

Thus, the decision to undergo screening can be complex and would depend on each individual patient’s preference [13, 14]. While the guidelines from the USPSTF and American College of Gynecologists and Obstetricians recommend breast cancer screening for all women up to 75 years old, the American Cancer Society recommends no longer screening once life expectancy is less than 10 years [14]. For women with ADRD aged 65 to 75 years, the estimated median survival time from ADRD diagnosis is 7.5 years [42], leading to conflicting recommendations from different guidelines. Strict adherence to clinical practice guidelines may lead to adverse events for older individuals with complex conditions [43]. Caregivers’ views toward cancer screening for ADRD patients may also vary by severity of dementia; caregivers of women with mild and moderate dementia are more likely to support continuation of mammogram than caregivers of women with severe dementia [44]. The impacts of ADRD on daily functioning and quality of life likely differ for each patient depending on the severity of dementia. This suggests that shared decision making about breast cancer screening is especially important for patients with ADRD, their caregivers, and providers.

Starting in 2020, the CMS quality metric for breast cancer screening has excluded elderly enrollees with both frailty and advanced illness, including dementia [7]. Future studies are needed to compare mammography rates before and after the CMS change to see if the issue of over-screening is diminished or eliminated in both MA and FFS for patients with ADRD. Despite these changes, patients with early-stage, less severe ADRD or cognitive impairment are unlikely to meet the frailty component of the exclusion criteria even when it may be appropriate to forego screening. Individuals with mild cognitive impairment or early-stage dementia often retain the decision-making capacity needed to be involved in their own health care decisions [45, 46]. Policymakers could consider allowing greater flexibility in how MA plans report preventive screening metrics for special populations such as those with less severe ADRD or cognitive impairment. Without those flexibilities, the existing reporting and bonus mechanisms of cancer screening may disadvantage certain MA plans, particularly those serving more ADRD patients in rural areas, despite the exclusions for advanced illness and frailty. Also, without those flexibilities, using cancer screening quality measures as a basis for health plan incentives or penalties may potentially lead to over-diagnosis and over-treatment in individuals with limited life expectancy.

### Limitations

This study has several limitations. First, given the limitations of the MCBS data, we were unable to observe several factors that may influence the decision to screen for breast cancer, including the severity of ADRD or cognitive impairment, social and mental health, and life expectancy [47, 48]. Although this study groups individuals with ADRD and cognitive impairment together, future studies should examine how cancer screening rates vary between these conditions and by disease severity. Second, this study was limited to older Medicare beneficiaries with ADRD or cognitive impairment. The findings may not be generalizable to those with early-onset ADRD. Third, self-reported or proxy-reported mammogram use may overestimate screening rates compared to claims-based estimates [37, 49]. Although MCBS interviewers encourage survey respondents to save documentation of their health care visits and services [19], the data for this study largely rely on the recall and executive functioning of Medicare beneficiaries with ADRD or cognitive impairment, who may have difficulty accurately recalling health care utilization. While the sensitivity analysis controlling for the use of proxy respondents produced the same results as the main analysis, the recall issue may remain. However, it is unlikely that any impaired recall of mammogram use systematically differs between FFS and MA enrollees.

## Conclusion

Our study found that Medicare beneficiaries age 65–74 with ADRD or cognitive impairment had a higher mammogram use rate when they were enrolled in MA plans compared to FFS Medicare, especially among those who live in urban areas and among dual-eligible enrollees. However, some Medicare Advantage enrollees with ADRD or cognitive impairment may have experienced over-screening for breast cancer. Future research should examine whether the changes in CMS screening measurements have reduced screening rates for vulnerable groups who are now excluded, such as those with ADRD.

### Electronic supplementary material

Below is the link to the electronic supplementary material.

Supplementary Material 1

## Data Availability

The data used in this study contain protected health information and are subject to a data use agreement with the Centers for Medicare & Medicaid Services, which restricts sharing the data. All our aggregated statistical results, including those unreported, are available to the public upon request to the corresponding author, Wendy Y. Xu.

## Abbreviations

ADRD: Alzheimer’s disease and related dementias
CMS: Centers for Medicare & Medicaid Services
FFS: Fee-for-service Medicare
MA: Medicare Advantage
MCBS: Medicare Current Beneficiary Survey
PR: Prevalence ratio
USPSTF: United States Preventive Services Task Force
