# Supplementary material for: Breast cancer screening among Medicare Advantage enrollees with dementia
Source: BMC Health Serv Res. 2024 Mar 5;24:283. doi: 10.1186/s12913-024-10740-7 (PMC10916275; doi:10.1186/s12913-024-10740-7)
Supplement: Supplementary file 1 — Supplementary Material 1 [file 12913_2024_10740_MOESM1_ESM.docx]

Supplemental File for “Breast cancer screening among Medicare Advantage enrollees with dementia”

| **Table S1.** Sample characteristics and unadjusted mammogram rates among Medicare enrollees age 75 years and older^a^ | | | | |
| --- | --- | --- | --- | --- |
|  | Sample Characteristics, % or mean (SD) | | Unadjusted Mammogram Rate,^b^ % | |
|  | Fee-for-Service Medicare | Medicare Advantage | Fee-for-Service Medicare | Medicare Advantage |
| Overall | -- | -- | 35.5 | 34.4 |
| Age in years, mean (SD) | 83.0 (5.9) | 82.4 (5.4) | -- | -- |
| Race / ethnicity | -- | -- | -- | -- |
| White (non-Hispanic), % | 82.5 | 78.3 | 35.7 | 32.2 |
| Black (non-Hispanic), % | 10.7 | 14.2 | 35.3 | 44.7 |
| Hispanic (any race), % | 3.1 | 4.5 | 35.9 | 43.3 |
| Asian, Native American, or other race (non-Hispanic), % | 3.7 | 3.0 | 33.0 | 29.2 |
| Urban residence, % | 74.4 | 86.1 | 35.6 | 34.4 |
| Rural residence, % | 25.6 | 13.9 | 35.4 | 34.2 |
| Dual-eligible, % | 22.3 | 26.3 | 30.7 | 31.5 |
| Non-dual-eligible, % | 77.7 | 73.7 | 36.9 | 35.4 |
| Number of body systems affected by comorbid conditions,^c^ mean (SD) | 4.5 (1.6) | 4.5 (1.6) | -- | -- |
| Cardiovascular disorder, % | 87.0 | 86.1 | 34.9 | 35.0 |
| Endocrine or metabolic disorder, % | 75.5 | 81.5 | 37.9 | 36.7 |
| Mental disorder, % | 42.1 | 42.6 | 38.3 | 36.1 |
| Musculoskeletal disorder, % | 77.1 | 71.1 | 37.3 | 37.1 |
| Neurological disorder, % | 5.8 | 6.6 | 31.2 | 48.2 |
| Respiratory disorder, % | 21.6 | 24.9 | 31.9 | 31.9 |
| Cancer, % | 32.7 | 27.3 | 39.3 | 37.2 |
| Vision impairment, % | 50.6 | 53.2 | 33.2 | 33.7 |
| Hearing impairment, % | 61.0 | 53.4 | 34.3 | 30.4 |
| Alzheimer’s disease and related dementias, % | 26.8 | 27.2 | 28.3 | 31.5 |
| Cognitive impairment, % | 73.2 | 72.8 | 38.2 | 35.4 |
| Income | -- | -- | -- | -- |
| Less than $15,000, % | 33.4 | 34.2 | 29.5 | 30.8 |
| $15,000 to $24,999, % | 25.4 | 28.1 | 30.7 | 33.6 |
| $25,000 to $49,999, % | 25.5 | 23.1 | 41.3 | 39.9 |
| $50,000 or more, % | 15.8 | 14.6 | 46.9 | 35.2 |
| Education | -- | -- | -- | -- |
| High school diploma or less, % | 61.8 | 64.2 | 32.5 | 31.3 |
| Vocational, technical, or business training, some college, or associate’s degree, % | 22.7 | 22.9 | 38.1 | 35.4 |
| Bachelor’s degree or higher, % | 15.6 | 12.9 | 43.8 | 47.8 |
| Proxy survey respondent, % | 18.1 | 11.1 | 24.0 | 15.6 |
|  |  |  |  |  |
| Survey respondents, n | 2378 | 1054 | -- | -- |
| Observations (person-year), n | 2980 | 1317 | -- | -- |
| Notes: ^a^Sample characteristics and unadjusted mammogram rates are weighted using Medicare Current Beneficiary Survey analytic weights.  ^b^The unadjusted mammogram rates are the proportion of observations in each enrollee characteristic group who reported receiving a biennial mammogram.  ^c^Comorbid conditions include self-reported cardiovascular disorders (arrhythmias, arteriosclerosis, coronary heart disease, heart failure, heart valve diseases, hypertension, or history of stroke), endocrine or metabolic disorders (diabetes, hyperlipidemia, or obesity), mental disorders (anxiety, depression, or other psychiatric disorder), musculoskeletal disorders (arthritis of any type, osteoporosis, or history of a broken hip), neurological disorders (Parkinson’s disease or paralysis) excluding Alzheimer’s disease and related dementias and cognitive impairment, respiratory disorders (asthma or chronic obstructive pulmonary disease), cancer (any type excluding breast cancer), vision impairment, and hearing impairment. | | | | |

| **Table S2**. Poisson regression model of mammogram utilization among Medicare enrollees age 75 years and older with Alzheimer’s disease and related dementias or cognitive impairment | | |
| --- | --- | --- |
|  | 75 and older sample (n=4297) | |
|  | Prevalence Ratio | 95% CI |
| Medicare enrollment (ref: fee-for-service Medicare) |  |  |
| Medicare Advantage | 0.94 | 0.84, 1.05 |
| Race/ethnicity (ref: White, non-Hispanic) |  |  |
| Black | 1.28 | 1.10, 1.48 |
| Hispanic | 1.47 | 1.12, 1.93 |
| Asian, Native American, or other race | 0.87 | 0.64, 1.18 |
| Residence (ref: urban) |  |  |
| Rural | 0.99 | 0.88, 1.11 |
| Age, years | 0.94 | 0.93, 0.95 |
| Number of body systems affected by comorbid conditions | 1.03 | 1.00, 1.07 |
| Dual eligibility (ref: no) |  |  |
| Yes | 0.90 | 0.77, 1.06 |
| Income (ref: $50,000 or more) |  |  |
| Less than $15,000 | 0.81 | 0.69, 0.94 |
| $15,000 to $24,999 | 0.88 | 0.77, 1.01 |
| $25,000 to $49,999 | 1.02 | 0.89, 1.17 |
| Education (ref: Bachelor’s degree or higher) |  |  |
| High school diploma or less | 0.79 | 0.70, 0.88 |
| Vocational, technical, or business training, some college, or associate’s degree | 0.86 | 0.74, 0.99 |

| **Table S3**. Poisson regression model of mammogram utilization among Medicare enrollees age 65-74 with Alzheimer’s disease and related dementias or cognitive impairment (including indicator for proxy survey respondent) | | |
| --- | --- | --- |
|  | Overall Sample (n=2090) | |
|  | Prevalence Ratio | 95% CI |
| Medicare enrollment (ref: fee-for-service Medicare) |  |  |
| Medicare Advantage | 1.16 | 1.05, 1.29 |
| Race/ethnicity (ref: White, non-Hispanic) |  |  |
| Black | 1.26 | 1.13, 1.42 |
| Hispanic | 1.32 | 1.10, 1.59 |
| Asian, Native American, or other race | 1.13 | 0.87, 1.46 |
| Residence (ref: urban) |  |  |
| Rural | 0.78 | 0.70, 0.87 |
| Age, years | 1.00 | 0.98, 1.02 |
| Number of body systems affected by comorbid conditions | 1.03 | 1.00, 1.06 |
| Dual eligibility (ref: no) |  |  |
| Yes | 0.91 | 0.79, 1.04 |
| Income (ref: $50,000 or more) |  |  |
| Less than $15,000 | 0.78 | 0.67, 0.90 |
| $15,000 to $24,999 | 0.79 | 0.69, 0.91 |
| $25,000 to $49,999 | 0.91 | 0.80, 1.02 |
| Education (ref: Bachelor’s degree or higher) |  |  |
| High school diploma or less | 0.91 | 0.79, 1.03 |
| Vocational, technical, or business training, some college, or associate’s degree | 0.84 | 0.72, 0.99 |
| Proxy survey respondent | 0.97 | 0.77, 1.21 |
